# The Role of *Kiss1* Neurons As Integrators of Endocrine, Metabolic, and Environmental Factors in the Hypothalamic–Pituitary–Gonadal Axis

**DOI:** 10.3389/fendo.2018.00188

**Published:** 2018-04-26

**Authors:** Shel-Hwa Yeo, William H. Colledge

**Affiliations:** Reproductive Physiology Group, Department of Physiology, Development and Neuroscience, University of Cambridge, Cambridge, United Kingdom

**Keywords:** *Kiss1*, hypothalamus, arcuate nucleus, gonadotropin-releasing hormone, neural afferents, luteinizing hormone

## Abstract

Kisspeptin–GPR54 signaling in the hypothalamus is required for reproduction and fertility in mammals. *Kiss1* neurons are key regulators of gonadotropin-releasing hormone (GnRH) release and modulation of the hypothalamic–pituitary–gonadal (HPG) axis. Arcuate *Kiss1* neurons project to GnRH nerve terminals in the median eminence, orchestrating the pulsatile secretion of luteinizing hormone (LH) through the intricate interaction between GnRH pulse frequency and the pituitary gonadotrophs. Arcuate *Kiss1* neurons, also known as KNDy neurons in rodents and ruminants because of their co-expression of neurokinin B and dynorphin represent an ideal hub to receive afferent inputs from other brain regions in response to physiological and environmental changes, which can regulate the HPG axis. This review will focus on studies performed primarily in rodent and ruminant species to explore potential afferent inputs to *Kiss1* neurons with emphasis on the arcuate region but also considering the rostral periventricular region of the third ventricle (RP3V). Specifically, we will discuss how these inputs can be modulated by hormonal, metabolic, and environmental factors to control gonadotropin secretion and fertility. We also summarize the methods and techniques that can be used to study functional inputs into *Kiss1* neurons.

## Introduction

Kisspeptin, encoded by the *Kiss1* gene, was initially proposed as a suppressor of metastasis, but its precise role in this process remains elusive (1, 2). Expression of the *KISS1* gene and the gene encoding its cognate receptor, the G-protein coupled receptor 54 (*GPR54*, also called *KISS1R*) has been detected in the brain and peripheral tissues, including the pancreas, liver, small intestine, pituitary, and placenta (1, 3, 4). Two seminal studies in 2003 defined a physiological role for kisspeptin by showing that inactivating mutations of the *GPR54* gene are associated with hypogonadotropic hypogonadism in humans and mice (5, 6). Kisspeptin signaling was thus identified as one of the critical regulators for both puberty onset and maintenance of normal reproductive functions in mammals (7, 8). Kisspeptin exerts its effects on the hypothalamic–pituitary–gonadal (HPG) axis, by acting as a neuropeptide essential for stimulation of gonadotropin-releasing hormone (GnRH) neurons in the hypothalamus (9–13). Subsequently, kisspeptin signaling has also been implicated in regulating glucose homeostasis and body weight control (14, 15).

This review focuses mainly on how *Kiss1* neurons in the arcuate region of the hypothalamus (*Kiss1^ARC^*) act as sensors to relay information about hormonal and nutritional status and environmental changes to GnRH neurons to regulate the secretion of the gonadotropins critical to sustain fertility (Table 1). It is important to decipher the upstream networks that connect to *Kiss1^ARC^* neurons, as this information will improve our understanding of how different cues can be integrated into the HPG axis, complementing the homeostatic regulation of reproductive function and maintaining fertility. Infertility is a global health issue affecting a significant proportion of humanity and is estimated to affect 8–12% of couples worldwide (16, 17). Given that kisspeptin has been successfully used in patients with hypothalamic amenorrhea (18, 19) as well as those with an absence of neurokinin B (NKB) signaling (20), insights into these central pathways will aid in the manipulation of kisspeptin signaling that may be used in the treatment of infertility and reproductive disorders.

**Table 1:** Summary of neuropeptides or hormones that interact with *Kiss1* neurons.

Candidates	Anatomical evidence to ARC or *Kiss1* neurons	ICV or electrophysiology studies	Receptors expressed in *Kiss1* neurons	Physiological relevance
Agouti-related peptide/neuropeptide Y (AgRP/NPY)	AgRP neurons formed synaptic connections with *Kiss1^ARC^* neurons (mice) (135)	Channel rhodopsin-assisted mapping indicated AgRP neurons formed inhibitory synaptic connections with *Kiss1^ARC^* neurons (mice) (135)	*Npy1r, Npy2r* and *Npy5r* (82)	Chemogenetic activation of AgRP neurons inhibited fertility *in vivo* (135)
Arginine vasopressin (AVP)	AVP-immunoreactive fibers in close apposition with *Kiss1^RP3V^* neurons (hamsters) (32)	AVP-stimulated *Kiss1^RP3V^* neurons (105)	*Avp1r* in *Kiss1^RP3V^* neurons (32, 105)	Circadian AVP signaling on *Kiss1^RP3V^* neurons is facilitated by estrogen during LH surge (32, 105)
Corticotropin-releasing hormone (CRH)	Close appositions of CRH-immunoreactive fibers and *Kiss1* neurons (125)	1. Central administration of CRH suppressed LH secretion in rats (117). 2. CRH stimulated LH and increased GnRH pulse amplitude in sheep (118, 119)	CRHR (125)	Possible regulator of GnRH secretion in stress-induced reproductive disorders
Dynorphin (DYN)	DYN-immunoreactive fibers in close apposition with *Kiss1^ARC^* neurons (rats and sheep) (171, 172)	ICV injection of dynorphin in adult male rats suppressed LH secretion (71)	*Oprk1* or KOR (39, 59, 73)	Inhibitory
Ghrelin		1. ICV administration inhibited LH secretion (rats and sheep) (143, 144). 2. Ghrelin acts on a subset of *Kiss1^ARC^* neurons and estradiol increases the sensitivity of these neurons to ghrelin signals (mice) (147)	*Ghsr or* GHSR1A (147)	Inhibitory
Leptin		ICV leptin treatment to fasted adult rats increased LH pulse frequency, amplitude, and mean levels (140)	*Lepr* (83, 141)	*Kiss1* neurons are indirect target of leptin during puberty onset (142)
Neurokinin B (NKB)	NKB-immunoreactive fibers in close apposition with *Kiss1^ARC^* neurons (rats and sheep) (65, 172)	NKB increased action potential firing of *Kiss1^ARC^* neurons *via* activation of NK3R (57–59)	NK1 tachykinin receptor (NK1R), NK2R and NK3R (58)	Stimulatory
Pituitary adenylate cyclase-activating peptide (PACAP)	Channel rhodopsin-assisted mapping indicated PACAP neurons from the PMV synapses onto *Kiss1* neurons (mice) (81)	1. ICV administered PACAP depressed plasma LH amplitude and pulse frequency in gonadectomized ewes (75). 2. PACAP elevated the plasma LH levels in male rats (76). 3. PMV PACAP neurons formed stimulatory synapses with *Kiss1* neurons (mice) (81)	*Adcyap1r1* (81, 82)	Critical for ovulatory cycling and fertility in females, acts as permissive role for leptin or nutritional regulation of reproductive function (81)
Proopiomelanocortin/Cocaine- and amphetamine-regulated transcript	Appositions between α-MSH fibers and *Kiss1^ARC^* neurons (rats and sheep) (131, 134)	Central alpha-MSH can stimulate or inhibit LH secretion in rats (129, 130)	MC4R (134)	*Kiss1^ARC^* neurons relay the stimulatory effects of melanocortin signaling onto the reproductive axis during puberty (134)
Relaxin-3	Dense relaxin-3-immunoreactive fibers projecting to the ARC (mice) (103)	ICV administration of human relaxin-3 in adult male rats stimulated LH secretion (102)	*Rxfp 1* (82)	Stimulatory
RFamide-related peptide-3 (RFRP-3)	RFRP-3-immunoreactive fibers contacted *Kiss1* neurons (mice and rats) (33, 90)	1. ICV RFRP-3 injection inhibits LH secretion in rats (91, 92), whereas it had no effects (97) in ewes. 2. Stimulates LH secretion in male hamsters (94, 95), but inhibits LH release in females (87, 93)	*Gpr147* or *Npffr1* (33, 90)	Primary central target for the inhibitory action of melatonin signal on reproductive function (98)
Somatostatin (SST)	Appositions between SST-immunoreactive fibers and *Kiss1* neurons (sheep) (85)	Inhibits episodic LH secretion during anestrus in sheep (84).	*Sstr1, 2, 3*, and *5* (82)	Inhibitory
Substance P (SP)	SP-immunoreactive fibers in close apposition with *Kiss1^ARC^* neurons (monkey) (70)	1. ICV administration of SP showed increased LH release (67). 2. SP activates *Kiss1^ARC^* neurons (58)	*Tacr1* or NK1R (68)	Stimulatory; critical in sustaining reproductive capabilities in female mice (66)

*Italic fonts indicated receptor gene expression identified by in situ hybridization or single cell reverse-transcription PCR. Receptors detected by immunostaining or pharmacological blockade were written in capital letters. ICV, intracerebroventricular; LH, luteinizing hormone; PMV, ventral premamillary nucleus of the hypothalamus; NK1, 2, and 3R, neurokinin-1-3 receptor; Sstr 1, 2, 3, and 5, somatostatin receptor 1, 2, 3, and 5; Gpr147, G protein-coupled receptor 147; Rxfp 1, relaxin/insulin like family peptide receptor 1; MC4R, melanocortin 4 receptor; Lepr, leptin receptor; Adcyap1r1, adenylate-cyclase-activating polypeptide 1 receptor 1; CRHR, corticotropin releasing hormone receptor; Npy1r, Npy2r, and Npy5r, neuropeptide Y receptor Y1, 2, and 5; Ghsr or GHSR1A, growth hormone secretagogue receptor type 1a; Npffr1, neuropeptide FF receptor 1; Oprk1 or KOR, opioid receptor, kappa 1; Tacr1, tachykinin receptor 1*.

## The Role of *Kiss1* Neurons in the Integration of Endocrine Responses

In mammals, there are two main populations of neurons in the hypothalamus that synthesize kisspeptin. The first is located in the preoptic area (POA) of sheep and monkeys (21–23), or the rostral periventricular region of the third ventricle (RP3V) in rodents (24, 25), hereafter, termed *Kiss1^RP3V^*. *Kiss1^RP3V^* neurons show a clear sexual dimorphism with greater numbers present in females (26) where they control the GnRH/LH surge that triggers ovulation (27). The *Kiss1^RP3V^* neurons express both estrogen receptor alpha (ERα) and progesterone receptor (27). Activation of *Kiss1^RP3V^* neurons by estradiol is essential for the positive feedback action of estrogen on the HPG axis and is associated with increased *Kiss1* mRNA expression (28). This is dependent on ERα because the increased firing of *Kiss1^RP3V^* neurons in response to estradiol is absent in ERα knockout mice (29–31). Peptides such as arginine vasopressin (AVP) (32) and gonadotropin-inhibitory hormone (GnIH) (33) also regulate *Kiss1^RP3V^* neurons. *Kiss1^RP3V^* neurons have been shown to co-express dopamine and galanin (34, 35). Neuroanatomical tracing using a recombinant adenovirus encoding farnesylated enhanced green fluorescent protein (EGFP) to facilitate the labeling of *Kiss1* neural axons revealed projection of *Kiss1^RP3V^* neurons to GnRH neuronal soma and proximal dendrites within the POA (36). The *Kiss1^RP3V^* neurons also project to the arcuate nucleus (ARC) and to the distal dendrites of GnRH neurons, suggesting that *Kiss1^RP3V^* and *Kiss1^ARC^* communicate with each other to synchronize or coordinate LH secretion (36).

A second population of *Kiss1* neurons, which are less well characterized, is found in the ARC region of the brain (also called the infundibular nucleus in humans and primates) (10, 21, 37, 38). In rodents, sheep, and goats, these neurons have been shown to co-express other neuropeptides such as NKB and dynorphin (DYN), and this has led to *Kiss1^ARC^* neurons being termed KNDy neurons (39–41). In KNDy neurons, estrogen suppresses *Kiss1* expression *via* ERα (28), and embryonic deletion of *Esr1* specifically in *Kiss1* neurons, advances puberty onset in association with significantly elevated LH levels (26, 42). *Kiss1^ARC^* neurons innervate the distal dendrons (a term describing a single projection structure that functions simultaneously as an axon and dendrite) (43) of GnRH neurons. Changes in *Kiss1^ARC^* neuron number, morphology, and connectivity with GnRH neurons have been detected across developmental stages (44–48).

## The Role of *Kiss1^ARC^* Neurons in Neuropeptide Integration

Optogenetic stimulation of *Kiss1^ARC^* neurons *in vivo* has shown an important role for these neurons in orchestrating pulsatile GnRH/LH secretion (49). Since then, the mechanism by which *Kiss1^ARC^* neurons contribute to the GnRH pulse generator has been studied. Navarro and colleagues proposed a model where *Kiss1^ARC^* neurons are interconnected and use the co-expressed neuropeptides NKB and DYN (39) to form a synchronized network sending a rhythmic stimulatory signal to the GnRH neurons, thus generating pulsatile gonadotropin secretion. Interestingly, optogenetic inhibition of *Kiss1^ARC^* neurons revealed that the middle or caudal *Kiss1^ARC^* neurons are responsible for pulsatile LH secretion whereas inhibition of rostral ARC failed to suppress LH release (50). The ARC population has been revealed to have higher number of *Kiss1* cells compared to the RP3V region (51, 52), and they may be heterogeneous in terms of firing pattern or ion channel distribution, and possibly receptor expression (53, 54). All in all, we are beginning to appreciate the complexity of the *Kiss1^ARC^* neurons in terms of their morphology, functional heterogeneity, different projection areas, and afferent inputs.

Central administration of various classical neurotransmitters and neuropeptides has been found to alter LH secretion through a GnRH neuron-dependent pathway (55). Given that *Kiss1^ARC^* neurons are upstream regulator of GnRH neurons, this indicates that *Kiss1^ARC^* neurons may possibly receive signals from a variety of neuropeptides and neuromodulators, which modulate GnRH/LH secretion by interacting with receptors located on kisspeptin neuron cell bodies, dendrites, and terminals. Another way to evaluate upstream signals to *Kiss1* neurons is to use electrophysiological recordings after neuropeptide stimulation in brain slice preparations and selective pharmacological inhibition of these responses. This approach was taken to show that *Kiss1* neurons can respond directly to NKB, DYN (56–58), and substance P (SP) (58). Once an initial firing response is identified, the next step is to undertake intracerebral injection or intracerebroventricular (ICV) infusion of the neuropeptide or antagonist *in vivo* to assess the corresponding physiological effects.

There is good evidence regarding the effects of the tachykinin NKB on *Kiss1^ARC^* neurons and subsequent LH release. *Kiss1^ARC^* neurons are depolarized and increase action potential firing upon activation of NK3R, the membrane receptor for NKB (57–59). In rodents, the full excitatory effect of NKB on *Kiss1^ARC^* neuron firing requires the activation of all three tachykinin receptor subtypes [NK1 tachykinin receptor (NK1R), NK2R and NK3R], which may all be expressed in these neurons (58). Activation of NK3R, with the agonist senktide, has been used in several studies to probe the effects of NKB on LH release in rodents (39, 60–62). The mechanisms of *Kiss1^ARC^* NKB signaling may vary between species as pulsatile LH release is sensitive to NK3R blockade alone in sheep (63), while in rodents, it requires blockade of all three tachykinin receptors with no effect with blockade of NK3R alone (64). The theory of a synchronized KNDy network as the pulse generator may be plausible; however, it is not clear whether these reciprocal KNDy–KNDy connections (65) derive from axon collaterals within a single neuron or connections from neighboring KNDy cells, or inputs from a segregated subset of KNDy neurons.

Substance P (SP) encoded by the *Tac1* gene, is another tachykinin, which can also influence reproduction. Female *Tac1* knockout mice display delayed puberty (66). Early studies with ovariectomized and estrogen-primed rats treated with intravenous or ICV administration of SP showed increased LH release (67). SP acts *via* the NK1R, and gonadotropin stimulation is blocked in the absence of kisspeptin (68). Moreover, SP activates *Kiss1^ARC^* neurons (58) and approximately half of the *Kiss1^ARC^* neurons express gene encoding the SP receptor (NK1R) (68). These findings support a role for SP acting *via Kiss1^ARC^* neurons to stimulate GnRH release and its critical role in sustaining reproductive capabilities in female mice. In rodents, populations of substance P cells were found in the ventromedial nucleus of the hypothalamus (VMH) (68, 69). One study reported SP fibers projection to the ARC, and they were in close apposition with *Kiss1^ARC^* neurons in male juvenile monkey (70). Majority of the SP cells were found in the premammillary nucleus, sparse SP cells were also observed in the ARC of the monkey.

Another substance that has been identified as an inhibitor of gonadotropin secretion is DYN (71), which belongs to the family of endogenous opioid peptides and is considered to mediate the negative feedback effects of progesterone on LH secretion (72). It has been reported that DYN exerts its inhibitory effects through *Kiss1* neurons (41) and the theory of DYN’s inhibitory effect on the GnRH pulse generator has emerged. More than 90% of *Kiss1^ARC^* neurons in the ewe express kappa opioid receptor (KOR) (73); whereas a lower percentage of KOR was revealed by *in situ* hybridization (39, 74) and single-cell reverse transcription-PCR studies (59). It is hypothesized that DYN may inhibit GnRH pulse frequency by binding to postsynaptic KOR in *Kiss1^ARC^* neurons. Further elucidation of the mechanism underlying DYN/KOR-dependent GnRH pulse generator suppression is yet to be proven.

The role of pituitary adenylate cyclase-activating peptide (PACAP) in puberty has been shown in several recent studies. In ovariectomized ewes, ICV administration of PACAP depressed plasma LH amplitude and pulse frequency (75). In contrast, intravenous infusion of PACAP elevated the plasma LH levels in male rats (76). Knockout of the PACAP gene (*Adcyap1)* is partially lethal (C57Bl/6J genetic background) as the majority of PACAP-deficient mice died at around 3 weeks of age (77) from dysfunction of lipid and carbohydrate metabolism (78). Surviving PACAP-deficient female mice exhibited reduced fertility, with no obvious defects in the length of estrus cycles but their mating frequency was significantly reduced (79). Abundant expression of *Adcyap1* is found in the ventral premammillary nucleus of the hypothalamus (PMV) and the VMH, both regions known to be involved in leptin-related control of puberty and fertility (80, 81). Despite the presence of leptin receptor (LepR) in *Kiss1^ARC^* neurons (82), the main site of leptin’s action to regulate reproduction is through cells in the PMV (83). Recently, channel rhodopsin-assisted circuit mapping revealed that the PMV PACAP neurons form direct monosynaptic contact with both *Kiss1^ARC^* and *Kiss1^RP3V^* neurons, which express the PACAP receptor (81). Furthermore, calcium-imaging experiments provided intriguing insights that PACAP exerts direct stimulatory effect exclusively on caudal *Kiss1^ARC^* neurons.

The peptide hormone somatostatin (SST), acting through the SSTR2 receptor, inhibits episodic LH secretion possibly *via* the mediobasal hypothalamus (MBH) during anestrus in sheep (84). Recently, Dufourny and Lomet discovered reciprocal connections between *Kiss1* and SST neurons in ewes (85), with most *Kiss1^ARC^* neurons showing SST-immunoreactive fiber appositions. The expression of *Sstr2* by *Kiss1* neurons is not yet proven but *Sstr2, 3*, and *4* are expressed in GnRH neurons in mice (86). SST neurons can be found in the periventricular area of the POA, in the ARC and in the ventrolateral area of the VMH. The functional relevance of *Kiss1* appositions on SST neurons remains to be verified since GPR54 has not yet been identified in SST neurons.

The RFamide-related peptide-3 (RFRP-3) is a mammalian analog of avian GnIH, found primarily in the dorsomedial nucleus of the hypothalamus (DMH) and adjacent structures (87, 88). This peptide inhibits LH secretion by suppressing GnRH secretion (89). Anatomical studies showed that about 20% of *Kiss1^RP3V^* neurons from proestrus female mice were contacted by RFRP-3 fibers, and only a small fraction of the *Kiss1^RP3V^* neurons expressed *Gpr147*, one of the receptors for RFRP-3 (33). Similarly, 35% of *Kiss1^ARC^* neurons receive RFRP fiber contacts, with approximately 25% expressed *Gpr147* (90). Even though RFRP-3 is considered an inhibitor of gonadotropin secretion in rats (91, 92) and female hamsters (87, 93), it is able to stimulate the gonadotropic axis in male Syrian and Siberian hamsters. RFRP-3′s stimulatory effects on gonadotropin and testosterone production were observed in male hamsters (94, 95). However, in the ewes, the initial inhibitory effects on LH secretion (96) was contradicted by a study performed by Decourt and colleagues (97), suggesting that RFRP-3 had no direct effect on LH release. Recently, RFRP-3 expressing neurons emerged as the primary central target for the inhibitory action of the melatonin signal after melatonin was reported have little or no effect on *Kiss1* neurons (98). RF-amide peptides are highly regulated by the melatonin-driven thyroid-stimulating hormone (TSH), which are critical for the control of seasonal breeding (99).

The neuropeptide oxytocin, which is involved in social bonding and sexual reproduction may also interact with *Kiss1* neurons. Central kisspeptin excitation of oxytocin neurons occurs in late pregnancy, and this excitation is likely to be mediated by a subpopulation of *Kiss1^RP3V^* projecting to the supraoptic nucleus (100). In contrast, the effect of oxytocin on *Kiss1^ARC^* neurons is unclear. One study revealed that intranasally applied oxytocin reaches the brain and oxytocin treatment at its highest dose increased the level of *Kiss1* and NKB mRNA in the anterior hypothalamus of female rats by approximately 400%, but *Kiss1* expression was unaffected by the lower doses of oxytocin. Also, elevated *Gnrh* mRNA expression following intranasally applied oxytocin was observed but the plasma LH concentration remained normal (101). *Kiss1* neurons have not been shown to express the oxytocin receptor; hence, the direct impact of oxytocin on *Kiss1* neurons within the anterior hypothalamus has yet to be confirmed.

McGowan and colleagues discovered a novel role for the neuropeptide relaxin-3 in the stimulation of the HPG axis *via* hypothalamic GnRH neurons (102), suggesting that this peptide may play a role in coordinating feeding and reproductive responses in response to alterations in energy balance. ICV administration of human relaxin-3 (H3) in adult male rats significantly increased plasma LH and the effect was blocked by pretreatment with a peripheral GnRH antagonist. H3 stimulated the release of GnRH from hypothalamic explants and GT1-7 cells, which express relaxin/insulin like family peptide receptor 1 and 3 (RXFP1 and RXFP3). Immunohistochemical labeling of relaxin-3-expressing neurons in male rats indicated dense relaxin-3-immunoreactive fibers projecting to the ARC. These relaxin-3-expressing cells were derived from the nucleus incertus, pontine raphe nucleus, periaqueductal gray, and the dorsal area to the substantia nigra detected by *in situ* hybridization (103). Further studies are required to define the physiological importance of relaxin-3 in the regulation of reproduction, and the possible interaction with *Kiss1^ARC^* neurons.

## *Kiss1* Neurons and the Integration of Environmental Cues

Many of the studies investigating *Kiss1^RP3V^* regulation have focused on factors that coordinate the LH surge with environmental cues, such as circadian inputs. Vasopressin (AVP) neurons from the suprachiasmatic nucleus (SCN) are the key SCN neurons regulating *Kiss1^RP3V^via* the V1a receptor and AVP-immunoreactive fibers project to *Kiss1^RP3V^* neurons (32). Vasopressin signaling is critically dependent on estrogen, in the presence of which vasopressin exerts a potent and direct stimulatory influence upon most *Kiss1^RP3V^* neurons (104, 105). Interestingly, estrogen permits circadian AVP signaling at *Kiss1^RP3V^* neurons without changes of AVP receptor signaling throughout the estrus cycle (105).

Seasonal breeders use photoperiod or day length as the primary environmental cue to time reproduction (106), ensuring birth occurs when environmental conditions favor the energetic demands of lactation and survival of offspring. The circulating levels of melatonin synchronize reproduction with photoperiod in these animals (98). These seasonal variations in reproduction are the direct result of changes in the neural network, specifically in (i) upstream neurons sensing the melatonin secretion from the pineal gland in response to photoperiod; (ii) neurons in the hypothalamus regulating GnRH and LH secretion (107). Ewes are short day breeders and remain in anestrus in long day conditions. Conversely, hamsters are long-day breeders (98). Both steroid-dependent and steroid-independent inhibition of gonadotropin secretion corresponding to photoperiod was demonstrated in sheep, Syrian and Siberian hamsters (108, 109). While *Kiss1^ARC^* neurons are the central site for the negative steroid feedback occurring in the breeding season, *Kiss1* expression is also inhibited by the short day melatonin signal (94, 110, 111). In the ewe, long days activate glutamatergic neurons that innervate A15 dopaminergic neurons in the retrochiasmatic area (112). Dopamine released from these neurons inhibits *Kiss1^ARC^* neuron activity, thus inhibiting GnRH/LH secretion and inducing infertility (113). In Syrian hamsters, long days stimulate the release of RFRP-3-expressing neurons in the DMH (114), which increases the activity of *Kiss1^ARC^* neurons. It is hypothesized that the increase in kisspeptin then stimulates GnRH secretion, causing testicular recrudescence and the resulting testosterone elevation stimulates *Kiss1^RP3V^* neurons (94). Conversely, kisspeptin appears to play no role in mediating the effects of photoperiod in male Siberian hamsters (115).

The hypothalamic paraventricular nucleus (PVN) contains a prominent population of corticotrophin-releasing factor (CRF) neurons, which regulate the hypothalamic–pituitary–adrenal axis (116). The role of PVN CRF in the control of LH secretion, however, is controversial even though it is known that stress responses can affect fertility. Central administration of CRH suppressed LH secretion in rats (117), whereas this is not the case in sheep (118, 119). Evidence of synaptic connections between CRF and GnRH neurons in the medial POA of rats (120) and the MBH of humans (121) indicate direct functional connection between CRF and GnRH neurons. Nevertheless, tract-tracing studies failed to find any CRF neurons projecting from the PVN to the POA where most GnRH neural soma are found in the rat (122). Stress-induced elevated CRF mRNA expression within the PVN did not correlate to LH pulse suppression (123) and finally, PVN lesions failed to interfere with the inhibitory effect of stress on LH release in rats (124). This suggests that CRF may act on one of the regulators of GnRH neurons rather than directly on GnRH neurons. Indeed, double-labeling immunohistochemistry revealed that most *Kiss1^RP3V^* and *Kiss1^ARC^* neurons in the female rat hypothalamus expressed the CRF receptor, CRHR. Close appositions of CRH-immunoreactive fibers on some of the *Kiss^RP3V^* and *Kiss1^ARC^n*eurons have been reported (125).

## Metabolic Integration *via Kiss1^ARC^* Neurons

Reproduction is normally coordinated with nutritional status to ensure that pregnancy, parturition, and lactation occur during periods of ample food to maximize the survival of the individual and their offspring (126). One way in which this is coordinated is *via* the anorexigenic and orexigenic actions of proopiomelanocortin (POMC) and agouti-related peptide (AgRP) neurons, respectively within the ARC on *Kiss1^ARC^* neurons. POMC and AgRP neurons are the first-order sensors of peripheral metabolic signals, such as the leptin and insulin to maintain energy homeostasis. The adipose tissue-derived hormone, leptin and stomach-derived ghrelin have also been implicated to exert effects on *Kiss1^ARC^* neurons.

### Alpha-Melanocyte-Stimulating (α-MSH) Hormone From POMC Neurons During Puberty

The role of α-MSH in the control of gonadotropin secretion in adults has been tested pharmacologically (127) and direct effects of α-MSH on GnRH neurons has been shown in adult mice (128). Central alpha-MSH can stimulate or inhibit LH secretion in rats depending on the hormonal milieu (129, 130). *Pomc* and *Kiss1* neurons appear to make mutual contacts in the adult ovine brain, α-MSH enhances *Kiss1* mRNA levels in the POA of sheep and decreases *Kiss1* expression in the ARC. Kisspeptin has been shown to inhibit *Pomc* gene expression in the ARC of the sheep (131), while ARC POMC neurons displayed increased firing after kisspeptin stimulation in mice (132). The cocaine- and amphetamine-regulated transcript (CART), another neuropeptide co-expressed in most POMC neurons can directly stimulate *Kiss1^ARC^* neurons in female mice (133), implying a possible functional connectivity between POMC and *Kiss1^ARC^* neurons. Manfredi-Lozano and teammates (134) performed a comprehensive pharmacogenetic and optogenetic approaches where using expression analyses, electrophysiological recordings, and a chemogenetic approach to pinpoint the physiological role of leptin acting *via* an α-MSH-kisspeptin pathway in the metabolic regulation of puberty. *Kiss1^ARC^* neurons appear to transmit the stimulatory effects of melanocortin signaling onto the reproductive axis during puberty based on these data: (1) presence of appositions between α-MSH fibers and *Kiss1^ARC^* neuronal cell bodies of pubertal female rats; (2) reduction of *Kiss1* mRNA expression in the ARC of pubertal females subjected to chronic inhibition of melanocortin 3 and 4 receptors (MC3/4R); (3) significant attenuation of LH responses to α-MSH in mice with congenital inactivation of *Gpr54*; and, importantly, (4) reduced LH responses to α-MSH following chemogenetic inhibition of *Kiss1^ARC^* neurons.

### Gamma-Aminobutyric Acid (GABA) From AgRP Neurons During Metabolic Deficiency

A seminal study by Padilla and colleagues indicated that AgRP-expressing neurons are activated during starvation and are involved in leptin-associated infertility during negative energy state (135). Using AgRP-neuron ablation and optogenetic strategies, they discovered inhibitory synaptic connections of AgRP neurons with neighboring *Kiss1^ARC^* neurons and rostral *Kiss1^RP3V^* neurons. The activated AgRP neurons release GABA, which has direct inhibitory actions on *Kiss1^ARC^* neurons. In agreement with this, *Kiss1^ARC^* neurons received less pre-synaptic inhibition in the absence of AgRP neurons after neonatal toxin-induced ablation. Chemogenetic activation of AgRP neurons as means of enhancing the activity of AgRP neurons over a sustained period is sufficient to perturb fertility *in vivo*. As a result, the animals exhibited delayed estrus cycles and decreased fertility (135). Interestingly, a direct, GABA-mediated connection between AgRP and GnRH neurons was not observed in this particular study despite the evidence that GnRH neurons are sensitive to a melanocortin agonist (136) and expressed NPY Y1 and Y5 (NPY1R and NPY5R*)* receptors (137). These findings confirmed that AgRP signaling contributes to infertility by inhibiting *Kiss1* during metabolic deficiency.

### Leptin and Ghrelin

Leptin deficiency is associated with suppressed *Kiss1* expression in rodents and sheep, while leptin administration has been shown to increase *Kiss1* expression (131, 138, 139) as well as elevating LH pulse frequency, amplitude, and mean levels (140). Although *Kiss1^ARC^* neurons express the LepR, only a small fraction of *Kiss1^ARC^* neurons are responsive to leptin (141) and deletion of LepRs from *Kiss1* neurons resulted in no puberty or fertility deficits (83). To eliminate the possibility of developmental adaptations and system redundancies, the LepR was selectively re-expressed in *Kiss1* neurons of *Lepr*-null mice. These mice showed no pubertal development and no improvement of the metabolic phenotype: they remained obese, diabetic, and infertile (142). These findings clearly demonstrate that *Kiss1* neurons are not the direct target of leptin during puberty onset. Cravo and colleagues also confirmed that leptin signaling in *Kiss1* neurons occurs only after completion of sexual development (142).

In addition to its undisputed role in the regulation of metabolism and energy balance, increasing evidence shows that ghrelin can influence fertility. Studies conducted in several species, including rats, sheep, and humans, indicate that ghrelin administration suppresses gonadotropin secretion (143–145). The ghrelin receptor, GHSR1a (growth hormone secretagogue receptor) is present in several hypothalamic regions, including those known to be involved in the control of the reproductive function, indicating that this hormone can interact directly with hypothalamic neurons (146). Work by Frazao and colleagues (147) confirmed that ghrelin interacts directly with a subpopulation of *Kiss1^ARC^* neurons to modulate their activity and that exposure to estradiol increases the sensitivity of these neurons to ghrelin signals. The effects of ghrelin varies according to the estrogen milieu, as it exerts a more pronounced orexigenic effect in ovariectomized female rats and diestrus females when estrogen levels are low. Males with estradiol treatment are resistant to the stimulatory effects of ghrelin on food intake (148). The physiological relevance of ghrelin effects on *Kiss1^ARC^* neurons in food intake and metabolic regulation requires further investigation.

## Experimental Approaches for Investigating Functional Inputs to *Kiss1^ARC^* Neurons

Recently, methodological advances have allowed us to gain significant insights into upstream signals that converge on *Kiss1* neurons to modulate the reproductive axis. One approach has been to use of single cell RNA sequencing (scRNA-seq) to identify the gene expression profile of *Kiss1* neurons and thereby identify the repertoire of surface receptor that may mediate physiological responses (82, 149). This approach provides the most direct and unbiased method to define a cell type based on its transcriptional profile, which can provide additional insights into connectivity and function (150, 151). Two groups recently carried out single cell analysis of neurons from the hypothalamus or the ARC that included *Kiss1^ARC^* neurons (82, 149). Results from these studies provide extensive information about the neuropeptides, neurotransmitters, and receptors co-expressed in *Kiss1^ARC^* neurons, facilitating the assessment of crosstalk among different neuropeptide signals within the same cell.

Both scRNA-seq studies revealed high expression of *Slc17a6*, which encodes a vesicular glutamate transporter and *Tac2* (NKB) in *Kiss1^ARC^* cells. In parallel, with previous data from electrophysiology, *in situ* hybridization and immunohistochemistry studies, *Kiss1^ARC^* neurons have been shown to express the NK3R (*Tacr3)*, estrogen receptor 1 (*Esr1*), receptors for progesterone, prolactin, ghrelin, and the neuropeptide FF receptor 1 (27, 28, 74, 147, 152, 153). In addition, the nociceptin receptor (*Oprl1)*, melanocortin receptors *(Mch3r and Mch4r)*, NPY receptors *(Npy1r, Npy2r* and *Npy5r)*, thyrotropin-releasing hormone receptor (*Trhr*), and insulin receptor substrate 4 (*Irs4*); are among the receptors that were identified in the *Kiss1^ARC^* single cells at low levels (149). These data suggest that *Kiss1^ARC^* neurons may receive afferent inputs from neurons involved in nociception, energy homeostasis, insulin signaling, and TSH secretion. Interestingly, given that *Kiss1^ARC^* single cells express four SST receptor subtypes (*Sstr1, 2, 3*, and *5*), the PACAP (*Adcyap1r1*), oxytocin (*Oxtr*), and RXFP1 (*Rxfp 1)* receptors (149), this information suggests a possible link between functional relevance of SST, PACAP, oxytocin, relaxin, and reproduction regulation *via Kiss1^ARC^* neurons, at least in females.

One limitation of the scRNA-seq studies is that the experiments are often designed to generate gene expression profiles without appropriate considerations of neuroendocrine criteria. Cell samples may be pooled from several animals, combining males and females, and for female samples; the stage of the estrus cycle may not be considered. Nevertheless, these data are still valid as preliminary information about the range of receptors expressed in the *Kiss1^ARC^* neural population. Specific scRNA-seq studies are necessary to further characterize the heterogeneity of the *Kiss1^ARC^* neurons.

Another approach to investigate functional inputs to *Kiss1^ARC^* neurons is to use powerful genetic methods in transgenic mice. The development of transgenic mouse lines with deletion of specific receptors in *Kiss1* neurons is a great tool in addressing the physiological relevance of these receptors. For example, mice have been generated in which the *Esr1* gene has been ablated in *Kiss1* neurons *via* a CRE-mediated recombination event (51), and these mice have defined the importance of sex steroid signaling in *Kiss1* neurons. Acute ablation of a gene from the earliest developmental time point, however, can sometimes be associated with compensatory changes in gene expression that can mask the effect of the gene disruption (154). If this occurs, a better alternative is to create a mouse model with inducible gene disruption to delineate the time windows in which gene inactivation is critical for the functional manifestation of a particular effect (155). Furthermore, varying the onset of gene manipulation at different time points and combining genetic manipulation with pharmacological or behavioral interventions will help to clarify gene–environment interactions that are crucial for the development or maintenance of reproductive phenotypes. The CRISPR/Cas9-based genome-editing tool implemented in mammalian cells has revolutionized gene-editing techniques (156). While this technique has generated huge impact on *in vitro* studies, progress is being made to also apply it *in vivo*. The combination of adeno-associated virus (AAV) and CRISPR/Cas9 system may be particularly useful in the future for editing reproductive-associated genes given that encouraging results have been obtained with the next-generation synthetic AAV capsids in several transgenic mouse models (157, 158).

A main objective in deciphering the neural circuitry is to define the synaptic inputs and outputs of specific neuronal subpopulations in different regions. Mapping the network of *Kiss1* neuronal inputs and outputs using a combination of molecular genetics and viral tract tracing techniques to provide both anatomical and functional circuit information is crucial. Until recently, the input–output relationships have been mapped using neuroanatomical tracers to reveal connections between regions (159). Classical tracers, such as biotin-dextran amine, fluorescent latex microspheres, fluorescent cholera toxin conjugates, or phaseolus vulgaris-leucoagglutinin, have provided very useful information to trace fibers in anterograde and retrograde directions, depending on the type of tracer applied (65, 160, 161), but they reveal only the axonal projections, not synaptic connections, and can be difficult to genetically target to specific neuronal types. Trans-synaptic tracing using retrograde viruses such as pseudorabies virus (PRV) is useful in revealing the brain regions forming synaptic connectivity with *Kiss1^ARC^* neurons (162), but this technique is only limited to rodents. The PRV is contagious to domestic mammals as it causes Aujeszsky disease in cattle and swines. However, the major drawback of this technique is that the PRV crosses multiple synapses, making it difficult to distinguish the first order and higher order synaptic inputs unless the PRV is combined with other neuronal tracers. To overcome the limitations of PRV tracing, monosynaptic tracing using glycoprotein (G)-deleted rabies virus is now a widely adopted method to delineate brain-wide monosynaptic connectivity (163).

Intensive efforts are being made to delineate the complete wiring diagram or connectome of the mammalian brain as a means to better understand how neural circuits control behavior. High-throughput electron microscopy has been used to delineate microscale connectivity (164), while tracing strategies utilizing viral tracers encoded with fluorophores have allowed for milliscale circuit mapping (165). These studies have elegantly dissected a number of complex circuits. However, these methodologies are not designed to provide molecular information about the pre-synaptic neural populations. These shortcomings warrant the identification of marker genes for neurons within the circuits to enable the testing of their functional role.

While neuroanatomical methods enable high-resolution mapping of neural circuitry, these approaches do not allow molecular profiling of neurons based on their connectivity. An advanced approach for translational profiling of neurons based on connectivity using viral translating ribosome affinity purification (vTRAP) has been reported recently (166). In this approach, CRE-dependent AAV or other retrograde viruses (rabies or canine adenovirus) are engineered to express an EGFP-tagged ribosome protein enabling isolation of mRNA that is being translated from a discrete CRE-expressing neural population. Projection-specific translational profiling is achieved by selectively precipitating neuronal ribosomes based on connectivity. Quantitative PCR is then used for selected target genes or high-throughput RNA sequencing to determine the neuronal identity without the need for detailed anatomical or electrophysiological investigation (166). The drawback of this technique is that high-throughput RNA sequencing on the immunoprecipitated RNA is critical and a substantial amount of validations is required prior to selection of appropriate marker genes for the projecting neurons.

An elegant study performed by Nectow and colleagues also used the vTRAP method to delineate the dorsal raphe nucleus (DRN) circuit in regulating feeding (167). First, they used an unbiased approach to map sites of neural activation in response to fasting, re-feeding, and hormonal cues, where subsets of DRN neurons were activated. Following this, comprehensive pharmacogenetic and optogenetic approaches were applied to carefully dissect the roles of DRN GABAergic neurons (DRN^Vgat^) and glutamatergic neurons (DRN^VGLUT3^) in modulating food intake. Transmembrane receptors that were enriched in both DRN^Vgat^ and DRN^VGLUT3^ neurons using the vTRAP method can be used for pharmacological screening of the ligands of these receptors. Finally, the effects of appropriate ligands for each receptor associated with the predicted response on feeding were tested using electrophysiological studies. This approach could also be used to molecularly characterize how the transcriptional profile of *Kiss1^ARC^* neurons changes in response to physiological stimuli.

Another powerful method to identify functional inputs into *Kiss1* neurons is to use optogenetic or chemogenetic approaches. These involve *a priori* identification of the specific pre-synaptic neural population as these methods required the use of neuron-specific CRE-expressing animal model. Both approaches involve the expression of non-native proteins that can function as channels (channel rhodopsin), pumps, or receptors in neurons. These novel proteins are sensitive only to exogenous non-native stimuli such as light or the compound clozapine-N-oxide (CNO). In this way, both approaches provide an exclusive selectivity for neuronal manipulations, enabling causal analyses of the roles of neural circuits in defined functions (168). Optogenetics allows millisecond-scale temporal accuracy in manipulating neuronal activity, which is critical for assessing circuit or behavioral functions that emphasizes on the rate or timing of neural activity. However, light activation of channel rhodopsin expressing neurons mainly release fast neurotransmitters such as glutamate and GABA; whereas neuropeptides require higher frequency and prolonged stimulations to be released. These factors may have explained the apparent lack of success for optogenetic release of neuropeptides (169). Alternatively, designer receptors exclusively activated by designer drugs that are sensitive to CNO and can be stimulated by a simple systemic CNO administration can be used. The main limitation of this approach is that their action is slow, in the order of minutes, rendering limited application in analyzing neural processes that rely on rate or timing of neural activity. But, this is ideal for examining the effects of chronic stimulation of neuronal populations.

To conclude, the plasticity or dynamics of the underlying kisspeptin–GnRH network in different physiological conditions is important. Relatively little is known about the role of kisspeptin under various physiological conditions such as negative energy state, lactation, and reproductive senescence. An excellent way to investigate the dynamics of the neural circuits connecting to *Kiss1^ARC^* neurons under these physiological conditions is to implement vTRAP on *Kiss1^ARC^* neurons followed by extensive chemogenetic and optogenetic strategies for causal analyses of the roles of specific neural circuits in defined behavioral responses or reproductive phenotypes.

## Concluding Remarks

Precise control of gonadotropin release by the HPG axis is essential for sustaining fertility in all mammals (170). Therefore, the HPG axis must be able to respond to changes in endocrine, metabolic, or environmental cues to regulate GnRH/LH release. The *Kiss1^ARC^* neurons are positioned as an ideal hub receiving afferent inputs from other brain regions in response to the internal homeostatic and external signal. Our understanding of the neural networks connecting with *Kiss1^ARC^* neurons is limited, however. To precisely identify and functionally characterize specific synaptic inputs to *Kiss1^ARC^* neurons poses a challenge, given that: (1) *Kiss1^ARC^* neurons are heterogeneous; (2) estrogen may have an organizational effect on the inputs; (3) there could be an interplay between the *Kiss1^RP3V^* and *Kiss1^ARC^* neurons in fine-tuning pulsatile GnRH/LH release. The search for afferent inputs into *Kiss1^ARC^* neurons is continuing using new technologies to decipher the neural network associated with the *Kiss1*-GnRH system. Techniques such as trans-synaptic viral tracing, single cell RNA sequencing combined with optogenetics and chemogenetics to allow functional analyses are providing significant knowledge about the regulation of *Kiss1^ARC^* neurons. The precise mechanisms delineating how neuropeptides/neuromodulators regulate *Kiss1^ARC^* neurons and fine-tune GnRH/LH secretion requires further characterization and validation. Also, careful considerations need to be implemented to distinguish direct actions of the neuropeptides on GnRH neurons or effects mediated through *Kiss1^ARC^* neurons.

## Author Contributions

S-HY wrote the review with input from WC.

## Conflict of Interest Statement

The authors declare that the research was conducted in the absence of any commercial or financial relationships that could be construed as a potential conflict of interest.

## References

[1] Ohtaki T Shintani Y Honda S Matsumoto H Hori A Kanehashi K Metastasis suppressor gene KiSS-1 encodes peptide ligand of a G-protein-coupled receptor. Nature (2001) 411:613–7. 10.1038/35079135 11385580

[2] Lee JH Miele ME Hicks DJ Phillips KK Trent JM Weissman BE KiSS-1, a novel human malignant melanoma metastasis-suppressor gene. J Natl Cancer Inst (1996) 88:1731–7. 10.1093/jnci/88.23.1731 8944003

[3] Gutierrez-Pascual E Martinez-Fuentes AJ Pinilla L Tena-Sempere M Malagon MM Castano JP. Direct pituitary effects of kisspeptin: activation of gonadotrophs and somatotrophs and stimulation of luteinising hormone and growth hormone secretion. J Neuroendocrinol (2007) 19:521–30. 10.1111/j.1365-2826.2007.01558.x 17532794

[4] Muir AI Chamberlain L Elshourbagy NA Michalovich D Moore DJ Calamari A AXOR12, a novel human G protein-coupled receptor, activated by the peptide KiSS-1. J Biol Chem (2001) 276:28969–75. 10.1074/jbc.M102743200 11387329

[5] de Roux N Genin E Carel JC Matsuda F Chaussain JL Milgrom E. Hypogonadotropic hypogonadism due to loss of function of the KiSS1-derived peptide receptor GPR54. Proc Natl Acad Sci U S A (2003) 100:10972–6. 10.1073/pnas.1834399100 12944565 PMC196911

[6] Seminara SB Messager S Chatzidaki EE Thresher RR Acierno JS Jr Shagoury JK The GPR54 gene as a regulator of puberty. N Engl J Med (2003) 349:1614–27. 10.1056/NEJMoa035322 14573733

[7] Herbison AE. Control of puberty onset and fertility by gonadotropin-releasing hormone neurons. Nat Rev Endocrinol (2016) 12:452–66. 10.1038/nrendo.2016.70 27199290

[8] d’Anglemont de Tassigny X Colledge WH The role of kisspeptin signaling in reproduction. Physiology (Bethesda) (2010) 25:207–17. 10.1152/physiol.00009.2010 20699467

[9] Navarro VM Castellano JM Fernandez-Fernandez R Barreiro ML Roa J Sanchez-Criado JE Developmental and hormonally regulated messenger ribonucleic acid expression of KiSS-1 and its putative receptor, GPR54, in rat hypothalamus and potent luteinizing hormone-releasing activity of KiSS-1 peptide. Endocrinology (2004) 145:4565–74. 10.1210/en.2004-0413 15242985

[10] Shahab M Mastronardi C Seminara SB Crowley WF Ojeda SR Plant TM. Increased hypothalamic GPR54 signaling: a potential mechanism for initiation of puberty in primates. Proc Natl Acad Sci U S A (2005) 102:2129–34. 10.1073/pnas.0409822102 15684075 PMC548549

[11] Irwig MS Fraley GS Smith JT Acohido BV Popa SM Cunningham MJ Kisspeptin activation of gonadotropin releasing hormone neurons and regulation of KiSS-1 mRNA in the male rat. Neuroendocrinology (2004) 80:264–72. 10.1159/000083140 15665556

[12] Kirilov M Clarkson J Liu X Roa J Campos P Porteous R Dependence of fertility on kisspeptin-Gpr54 signaling at the GnRH neuron. Nat Commun (2013) 4:2492. 10.1038/ncomms3492 24051579

[13] Wen S Ai W Alim Z Boehm U. Embryonic gonadotropin-releasing hormone signaling is necessary for maturation of the male reproductive axis. Proc Natl Acad Sci U S A (2010) 107:16372–7. 10.1073/pnas.1000423107 20805495 PMC2941299

[14] Song WJ Mondal P Wolfe A Alonso LC Stamateris R Ong BW Glucagon regulates hepatic kisspeptin to impair insulin secretion. Cell Metab (2014) 19:667–81. 10.1016/j.cmet.2014.03.005 24703698 PMC4058888

[15] Tolson KP Garcia C Yen S Simonds S Stefanidis A Lawrence A Impaired kisspeptin signaling decreases metabolism and promotes glucose intolerance and obesity. J Clin Invest (2014) 124:3075–9. 10.1172/JCI71075 24937427 PMC4071390

[16] Ombelet W Cooke I Dyer S Serour G Devroey P. Infertility and the provision of infertility medical services in developing countries. Hum Reprod Update (2008) 14:605–21. 10.1093/humupd/dmn042 18820005 PMC2569858

[17] Inhorn MC Patrizio P. Infertility around the globe: new thinking on gender, reproductive technologies and global movements in the 21st century. Hum Reprod Update (2015) 21:411–26. 10.1093/humupd/dmv016 25801630

[18] Jayasena CN Nijher GM Chaudhri OB Murphy KG Ranger A Lim A Subcutaneous injection of kisspeptin-54 acutely stimulates gonadotropin secretion in women with hypothalamic amenorrhea, but chronic administration causes tachyphylaxis. J Clin Endocrinol Metab (2009) 94:4315–23. 10.1210/jc.2009-0406 19820030

[19] Jayasena CN Abbara A Veldhuis JD Comninos AN Ratnasabapathy R De Silva A Increasing LH pulsatility in women with hypothalamic amenorrhoea using intravenous infusion of Kisspeptin-54. J Clin Endocrinol Metab (2014) 99:E953–61. 10.1210/jc.2013-1569 24517142 PMC4207927

[20] Young J George JT Tello JA Francou B Bouligand J Guiochon-Mantel A Kisspeptin restores pulsatile LH secretion in patients with neurokinin B signaling deficiencies: physiological, pathophysiological and therapeutic implications. Neuroendocrinology (2013) 97:193–202. 10.1159/000336376 22377698 PMC3902960

[21] Estrada KM Clay CM Pompolo S Smith JT Clarke IJ. Elevated KiSS-1 expression in the arcuate nucleus prior to the cyclic preovulatory gonadotrophin-releasing hormone/lutenising hormone surge in the ewe suggests a stimulatory role for kisspeptin in oestrogen-positive feedback. J Neuroendocrinol (2006) 18:806–9. 10.1111/j.1365-2826.2006.01485.x 16965299

[22] Smith JT Clay CM Caraty A Clarke IJ. KiSS-1 messenger ribonucleic acid expression in the hypothalamus of the ewe is regulated by sex steroids and season. Endocrinology (2007) 148:1150–7. 10.1210/en.2006-1435 17185374

[23] Smith JT Shahab M Pereira A Pau KY Clarke IJ. Hypothalamic expression of KISS1 and gonadotropin inhibitory hormone genes during the menstrual cycle of a non-human primate. Biol Reprod (2010) 83:568–77. 10.1095/biolreprod.110.085407 20574054 PMC2957156

[24] Clarkson J dAnglemont de Tassigny X Colledge WH Caraty A Herbison AE. Distribution of kisspeptin neurones in the adult female mouse brain. J Neuroendocrinol (2009) 21:673–82. 10.1111/j.1365-2826.2009.01892.x 19515163

[25] Adachi S Yamada S Takatsu Y Matsui H Kinoshita M Takase K Involvement of anteroventral periventricular metastin/kisspeptin neurons in estrogen positive feedback action on luteinizing hormone release in female rats. J Reprod Dev (2007) 53:367–78. 10.1262/jrd.18146 17213691

[26] Kauffman AS Gottsch ML Roa J Byquist AC Crown A Clifton DK Sexual differentiation of Kiss1 gene expression in the brain of the rat. Endocrinology (2007) 148:1774–83. 10.1210/en.2006-1540 17204549

[27] Clarkson J dAnglemont de Tassigny X Moreno AS Colledge WH Herbison AE. Kisspeptin-GPR54 signaling is essential for preovulatory gonadotropin-releasing hormone neuron activation and the luteinizing hormone surge. J Neurosci (2008) 28:8691–7. 10.1523/JNEUROSCI.1775-08.2008 18753370 PMC6670827

[28] Smith JT Cunningham MJ Rissman EF Clifton DK Steiner RA. Regulation of Kiss1 gene expression in the brain of the female mouse. Endocrinology (2005) 146:3686–92. 10.1210/en.2005-0323 15919741

[29] Gottsch ML Navarro VM Zhao Z Glidewell-Kenney C Weiss J Jameson JL Regulation of Kiss1 and dynorphin gene expression in the murine brain by classical and nonclassical estrogen receptor pathways. J Neurosci (2009) 29:9390–5. 10.1523/JNEUROSCI.0763-09.2009 19625529 PMC2819182

[30] Frazao R Cravo RM Donato J Jr Ratra DV Clegg DJ Elmquist JK Shift in Kiss1 cell activity requires estrogen receptor alpha. J Neurosci (2013) 33:2807–20. 10.1523/JNEUROSCI.1610-12.2013 23407940 PMC3713640

[31] Dubois SL Acosta-Martinez M DeJoseph MR Wolfe A Radovick S Boehm U Positive, but not negative feedback actions of estradiol in adult female mice require estrogen receptor alpha in kisspeptin neurons. Endocrinology (2015) 156:1111–20. 10.1210/en.2014-1851 25545386 PMC4330313

[32] Williams WP III Jarjisian SG Mikkelsen JD Kriegsfeld LJ. Circadian control of kisspeptin and a gated GnRH response mediate the preovulatory luteinizing hormone surge. Endocrinology (2011) 152:595–606. 10.1210/en.2010-0943 21190958 PMC3037169

[33] Rizwan MZ Poling MC Corr M Cornes PA Augustine RA Quennell JH RFamide-related peptide-3 receptor gene expression in GnRH and kisspeptin neurons and GnRH-dependent mechanism of action. Endocrinology (2012) 153:3770–9. 10.1210/en.2012-1133 22691552

[34] Clarkson J Herbison AE. Dual phenotype kisspeptin-dopamine neurones of the rostral periventricular area of the third ventricle project to gonadotrophin-releasing hormone neurones. J Neuroendocrinol (2011) 23:293–301. 10.1111/j.1365-2826.2011.02107.x 21219482

[35] Porteous R Petersen SL Yeo SH Bhattarai JP Ciofi P de Tassigny XD Kisspeptin neurons co-express met-enkephalin and galanin in the rostral periventricular region of the female mouse hypothalamus. J Comp Neurol (2011) 519:3456–69. 10.1002/cne.22716 21800299

[36] Yip SH Boehm U Herbison AE Campbell RE Conditional viral tract-tracing delineates the projections of the distinct kisspeptin neuron populations to gonadotropin-releasing hormone (GnRH) neurons in the mouse. Endocrinology (2015) 156:2582–94. 10.1210/en.2015-1131 25856430

[37] Franceschini I Lomet D Cateau M Delsol G Tillet Y Caraty A. Kisspeptin immunoreactive cells of the ovine preoptic area and arcuate nucleus co-express estrogen receptor alpha. Neurosci Lett (2006) 401:225–30. 10.1016/j.neulet.2006.03.039 16621281

[38] Gottsch ML Cunningham MJ Smith JT Popa SM Acohido BV Crowley WF A role for kisspeptins in the regulation of gonadotropin secretion in the mouse. Endocrinology (2004) 145:4073–7. 10.1210/en.2004-0431 15217982

[39] Navarro VM Gottsch ML Chavkin C Okamura H Clifton DK Steiner RA. Regulation of gonadotropin-releasing hormone secretion by kisspeptin/dynorphin/neurokinin B neurons in the arcuate nucleus of the mouse. J Neurosci (2009) 29:11859–66. 10.1523/JNEUROSCI.1569-09.2009 19776272 PMC2793332

[40] Goodman RL Lehman MN Smith JT Coolen LM de Oliveira CV Jafarzadehshirazi MR Kisspeptin neurons in the arcuate nucleus of the ewe express both dynorphin A and neurokinin B. Endocrinology (2007) 148:5752–60. 10.1210/en.2007-0961 17823266

[41] Wakabayashi Y Nakada T Murata K Ohkura S Mogi K Navarro VM Neurokinin B and dynorphin A in kisspeptin neurons of the arcuate nucleus participate in generation of periodic oscillation of neural activity driving pulsatile gonadotropin-releasing hormone secretion in the goat. J Neurosci (2010) 30:3124–32. 10.1523/JNEUROSCI.5848-09.2010 20181609 PMC6633939

[42] Kauffman AS Navarro VM Kim J Clifton D Steiner RA. Sex differences in the regulation of Kiss1/NKB neurons in juvenile mice: implications for the timing of puberty. Am J Physiol Endocrinol Metab (2009) 297:E1212–21. 10.1152/ajpendo.00461.2009 19755669 PMC2781353

[43] Herde MK Iremonger KJ Constantin S Herbison AE. GnRH neurons elaborate a long-range projection with shared axonal and dendritic functions. J Neurosci (2013) 33:12689–97. 10.1523/JNEUROSCI.0579-13.2013 23904605 PMC6618539

[44] Kumar D Freese M Drexler D Hermans-Borgmeyer I Marquardt A Boehm U. Murine arcuate nucleus kisspeptin neurons communicate with GnRH neurons in utero. J Neurosci (2014) 34:3756–66. 10.1523/JNEUROSCI.5123-13.2014 24599473 PMC6608990

[45] Desroziers E Mikkelsen JD Duittoz A Franceschini I. Kisspeptin-immunoreactivity changes in a sex- and hypothalamic-region-specific manner across rat postnatal development. J Neuroendocrinol (2012) 24:1154–65. 10.1111/j.1365-2826.2012.02317.x 22458373

[46] Semaan SJ Tolson KP Kauffman AS. The development of kisspeptin circuits in the mammalian brain. Adv Exp Med Biol (2013) 784:221–52. 10.1007/978-1-4614-6199-9_11 23550009 PMC4305450

[47] Lehman MN Hileman SM Goodman RL. Neuroanatomy of the kisspeptin signaling system in mammals: comparative and developmental aspects. Adv Exp Med Biol (2013) 784:27–62. 10.1007/978-1-4614-6199-9_3 23550001 PMC4059209

[48] Takumi K Iijima N Ozawa H. Developmental changes in the expression of kisspeptin mRNA in rat hypothalamus. J Mol Neurosci (2011) 43:138–45. 10.1007/s12031-010-9430-1 20665248

[49] Han SY McLennan T Czieselsky K Herbison AE. Selective optogenetic activation of arcuate kisspeptin neurons generates pulsatile luteinizing hormone secretion. Proc Natl Acad Sci U S A (2015) 112:13109–14. 10.1073/pnas.1512243112 26443858 PMC4620857

[50] Clarkson J Han SY Piet R McLennan T Kane GM Ng J Definition of the hypothalamic GnRH pulse generator in mice. Proc Natl Acad Sci U S A (2017) 114:E10216–23. 10.1073/pnas.1713897114 29109258 PMC5703322

[51] Mayer C Acosta-Martinez M Dubois SL Wolfe A Radovick S Boehm U Timing and completion of puberty in female mice depend on estrogen receptor alpha-signaling in kisspeptin neurons. Proc Natl Acad Sci U S A (2010) 107:22693–8. 10.1073/pnas.1012406108 21149719 PMC3012491

[52] Yeo SH Kyle V Morris PG Jackman S Sinnett-Smith LC Schacker M Visualisation of Kiss1 neurone distribution using a Kiss1-CRE transgenic mouse. J Neuroendocrinol (2016) 28(11). 10.1111/jne.12435 27663274 PMC5091624

[53] Piet R de Croft S Liu X Herbison AE. Electrical properties of kisspeptin neurons and their regulation of GnRH neurons. Front Neuroendocrinol (2015) 36C:15–27. 10.1016/j.yfrne.2014.05.006 24907402

[54] Mendonca PRF Kyle V Yeo SH Colledge WH Robinson HPC Kv4.2 channel activity controls intrinsic firing dynamics of arcuate kisspeptin neurons. J Physiol (2017) 596:885–99. 10.1113/JP274474 PMC5830417 29214635

[55] Herbison A Physiology of the gonadotropin-releasing hormone neuronal network. Knobil and Neills Physiology of Reproduction. San Diego: Elsiever (2006) 1:1415–82.

[56] Navarro VM Castellano JM McConkey SM Pineda R Ruiz-Pino F Pinilla L Interactions between kisspeptin and neurokinin B in the control of GnRH secretion in the female rat. Am J Physiol Endocrinol Metab (2011) 300:E202–10. 10.1152/ajpendo.00517.2010 21045176 PMC3774070

[57] Gottsch ML Popa SM Lawhorn JK Qiu J Tonsfeldt KJ Bosch MA Molecular properties of kiss1 neurons in the arcuate nucleus of the mouse. Endocrinology (2011) 152:4298–309. 10.1210/en.2011-1521 21933870 PMC3199004

[58] de Croft S Boehm U Herbison AE. Neurokinin B activates arcuate kisspeptin neurons through multiple tachykinin receptors in the male mouse. Endocrinology (2013) 154:2750–60. 10.1210/en.2013-1231 23744641

[59] Ruka KA Burger LL Moenter SM Regulation of arcuate neurons coexpressing kisspeptin, neurokinin B, and dynorphin by modulators of neurokinin 3 and kappa-opioid receptors in adult male mice. Endocrinology (2013) 154:2761–71. 10.1210/en.2013-1268 23744642 PMC3713217

[60] Garcia-Galiano D van Ingen Schenau D Leon S Krajnc-Franken MA Manfredi-Lozano M Romero-Ruiz A Kisspeptin signaling is indispensable for neurokinin B, but not glutamate, stimulation of gonadotropin secretion in mice. Endocrinology (2012) 153:316–28. 10.1210/en.2011-1260 22067321

[61] Grachev P Li XF Kinsey-Jones JS di Domenico AL Millar RP Lightman SL Suppression of the GnRH pulse generator by neurokinin B involves a kappa-opioid receptor-dependent mechanism. Endocrinology (2012) 153:4894–904. 10.1210/en.2012-1574 22903614

[62] Kinsey-Jones JS Grachev P Li XF Lin YS Milligan SR Lightman SL The inhibitory effects of neurokinin B on GnRH pulse generator frequency in the female rat. Endocrinology (2012) 153:307–15. 10.1210/en.2011-1641 22109887

[63] Goodman RL Hileman SM Nestor CC Porter KL Connors JM Hardy SL Kisspeptin, neurokinin B, and dynorphin act in the arcuate nucleus to control activity of the GnRH pulse generator in ewes. Endocrinology (2013) 154:4259–69. 10.1210/en.2013-1331 23959940 PMC3800763

[64] Noritake K Matsuoka T Ohsawa T Shimomura K Sanbuissho A Uenoyama Y Involvement of neurokinin receptors in the control of pulsatile luteinizing hormone secretion in rats. J Reprod Dev (2011) 57:409–15. 10.1262/jrd.11-002S 21358144

[65] Krajewski SJ Burke MC Anderson MJ McMullen NT Rance NE. Forebrain projections of arcuate neurokinin B neurons demonstrated by anterograde tract-tracing and monosodium glutamate lesions in the rat. Neuroscience (2010) 166:680–97. 10.1016/j.neuroscience.2009.12.053 20038444 PMC2823949

[66] Simavli S Thompson IR Maguire CA Gill JC Carroll RS Wolfe A Substance p regulates puberty onset and fertility in the female mouse. Endocrinology (2015) 156:2313–22. 10.1210/en.2014-2012 25856429 PMC4430622

[67] Arisawa M De Palatis L Ho R Snyder GD Yu WH Pan G Stimulatory role of substance P on gonadotropin release in ovariectomized rats. Neuroendocrinology (1990) 51:523–9. 10.1159/000125386 1693756

[68] Navarro VM Bosch MA Leon S Simavli S True C Pinilla L The integrated hypothalamic tachykinin-kisspeptin system as a central coordinator for reproduction. Endocrinology (2015) 156:627–37. 10.1210/en.2014-1651 25422875 PMC4298326

[69] Ljungdahl A Hokfelt T Nilsson G Goldstein M Distribution of substance P-like immunoreactivity in the central nervous system of the rat – II. Light microscopic localization in relation to catecholamine-containing neurons. Neuroscience (1978) 3:945–76. 10.1016/0306-4522(78)90117-3 32496

[70] Kalil B Ramaswamy S Plant TM. The distribution of substance P and kisspeptin in the mediobasal hypothalamus of the male rhesus monkey and a comparison of intravenous administration of these peptides to release GnRH as reflected by LH secretion. Neuroendocrinology (2016) 103:711–23. 10.1159/000442420 26580201 PMC4873470

[71] Kinoshita F Nakai Y Katakami H Imura H. Suppressive effect of dynorphin-(1-13) on luteinizing hormone release in conscious castrated rats. Life Sci (1982) 30:1915–9. 10.1016/0024-3205(82)90472-6 6287134

[72] Goodman RL Coolen LM Anderson GM Hardy SL Valent M Connors JM Evidence that dynorphin plays a major role in mediating progesterone negative feedback on gonadotropin-releasing hormone neurons in sheep. Endocrinology (2004) 145:2959–67. 10.1210/en.2003-1305 14988383

[73] Weems PW Witty CF Amstalden M Coolen LM Goodman RL Lehman MN kappa-opioid receptor is colocalized in GnRH and KNDy cells in the female ovine and rat brain. Endocrinology (2016) 157:2367–79. 10.1210/en.2015-1763 27064940 PMC4891780

[74] Navarro VM Gottsch ML Wu M Garcia-Galiano D Hobbs SJ Bosch MA Regulation of NKB pathways and their roles in the control of Kiss1 neurons in the arcuate nucleus of the male mouse. Endocrinology (2011) 152:4265–75. 10.1210/en.2011-1143 21914775 PMC3198996

[75] Sawangjaroen K Curlewis JD. Effects of pituitary adenylate cyclase-activating polypeptide (PACAP) and vasoactive intestinal polypeptide (VIP) on prolactin, luteinizing hormone and growth hormone secretion in the ewe. J Neuroendocrinol (1994) 6:549–55. 10.1111/j.1365-2826.1994.tb00618.x 7827625

[76] Koch B Lutz-Bucher B. Pituitary adenylate cyclase-activating polypeptide (PACAP) stimulates cyclic AMP formation as well as peptide output of cultured pituitary melanotrophs and AtT-20 corticotrophs. Regul Pept (1992) 38:45–53. 10.1016/0167-0115(92)90071-2 1315448

[77] Hashimoto H Shintani N Tanaka K Mori W Hirose M Matsuda T Altered psychomotor behaviors in mice lacking pituitary adenylate cyclase-activating polypeptide (PACAP). Proc Natl Acad Sci U S A (2001) 98:13355–60. 10.1073/pnas.231094498 11687615 PMC60875

[78] Gray SL Cummings KJ Jirik FR Sherwood NM. Targeted disruption of the pituitary adenylate cyclase-activating polypeptide gene results in early postnatal death associated with dysfunction of lipid and carbohydrate metabolism. Mol Endocrinol (2001) 15:1739–47. 10.1210/mend.15.10.0705 11579206

[79] Szabo E Nemeskeri A Arimura A Koves K. Effect of PACAP on LH release studied by cell immunoblot assay depends on the gender, on the time of day and in female rats on the day of the estrous cycle. Regul Pept (2004) 123:139–45. 10.1016/j.regpep.2004.04.021 15518904

[80] Ross RA Maguire CA Verstegen AMJ Kaiser UB Lowell BB Navarro VM The effect of PACAP on fertility is relayed through a subset of hypothalamic leptin receptor expressing neurons in the female mouse. Endocrine Societys 98th Annual Meeting and Expo; 2016 Apr 1–4; Boston (2016).

[81] Ross RA León S Madara JC Schafer D Fergani C Maguire CA PACAP neurons in the ventral premammillary nucleus regulate reproductive function in the female mouse. (2018). 10.1101/274860 PMC6013253 29905528

[82] Campbell JN Macosko EZ Fenselau H Pers TH Lyubetskaya A Tenen D A molecular census of arcuate hypothalamus and median eminence cell types. Nat Neurosci (2017) 20:484–96. 10.1038/nn.4495 28166221 PMC5323293

[83] Donato J Jr Cravo RM Frazao R Gautron L Scott MM Lachey J Leptins effect on puberty in mice is relayed by the ventral premammillary nucleus and does not require signaling in Kiss1 neurons. J Clin Invest (2011) 121:355–68. 10.1172/JCI45106 21183787 PMC3007164

[84] McCosh RB Szeligo BM Bedenbaugh MN Lopez JA Hardy SL Hileman SM Evidence that endogenous somatostatin inhibits episodic, but not surge, secretion of LH in female sheep. Endocrinology (2017) 158:1827–37. 10.1210/en.2017-00075 28379327 PMC5460938

[85] Dufourny L Lomet D. Crosstalks between kisspeptin neurons and somatostatin neurons are not photoperiod dependent in the ewe hypothalamus. Gen Comp Endocrinol (2017) 254:68–74. 10.1016/j.ygcen.2017.09.017 28935581

[86] Todman MG Han SK Herbison AE. Profiling neurotransmitter receptor expression in mouse gonadotropin-releasing hormone neurons using green fluorescent protein-promoter transgenics and microarrays. Neuroscience (2005) 132:703–12. 10.1016/j.neuroscience.2005.01.035 15837132

[87] Kriegsfeld LJ Mei DF Bentley GE Ubuka T Mason AO Inoue K Identification and characterization of a gonadotropin-inhibitory system in the brains of mammals. Proc Natl Acad Sci U S A (2006) 103:2410–5. 10.1073/pnas.0511003103 16467147 PMC1413747

[88] Dardente H Birnie M Lincoln GA Hazlerigg DG. RFamide-related peptide and its cognate receptor in the sheep: cDNA cloning, mRNA distribution in the hypothalamus and the effect of photoperiod. J Neuroendocrinol (2008) 20:1252–9. 10.1111/j.1365-2826.2008.01784.x 18752651

[89] Smith JT Coolen LM Kriegsfeld LJ Sari IP Jaafarzadehshirazi MR Maltby M Variation in kisspeptin and RFamide-related peptide (RFRP) expression and terminal connections to gonadotropin-releasing hormone neurons in the brain: a novel medium for seasonal breeding in the sheep. Endocrinology (2008) 149:5770–82. 10.1210/en.2008-0581 18617612 PMC2584593

[90] Poling MC Quennell JH Anderson GM Kauffman AS Kisspeptin neurones do not directly signal to RFRP-3 neurones but RFRP-3 may directly modulate a subset of hypothalamic kisspeptin cells in mice. J Neuroendocrinol (2013) 25:876–86. 10.1111/jne.12084 23927071 PMC4022484

[91] Pineda R Garcia-Galiano D Sanchez-Garrido MA Romero M Ruiz-Pino F Aguilar E Characterization of the inhibitory roles of RFRP3, the mammalian ortholog of GnIH, in the control of gonadotropin secretion in the rat: in vivo and in vitro studies. Am J Physiol Endocrinol Metab (2010) 299:E39–46. 10.1152/ajpendo.00108.2010 20424142

[92] Murakami M Matsuzaki T Iwasa T Yasui T Irahara M Osugi T Hypophysiotropic role of RFamide-related peptide-3 in the inhibition of LH secretion in female rats. J Endocrinol (2008) 199:105–12. 10.1677/JOE-08-0197 18653621

[93] Henningsen JB Ancel C Mikkelsen JD Gauer F Simonneaux V. Roles of RFRP-3 in the daily and seasonal regulation of reproductive activity in female Syrian hamsters. Endocrinology (2017) 158:652–63. 10.1210/en.2016-1689 27983867

[94] Ancel C Bentsen AH Sebert ME Tena-Sempere M Mikkelsen JD Simonneaux V. Stimulatory effect of RFRP-3 on the gonadotrophic axis in the male Syrian hamster: the exception proves the rule. Endocrinology (2012) 153:1352–63. 10.1210/en.2011-1622 22275511

[95] Ubuka T Inoue K Fukuda Y Mizuno T Ukena K Kriegsfeld LJ Identification, expression, and physiological functions of Siberian hamster gonadotropin-inhibitory hormone. Endocrinology (2012) 153:373–85. 10.1210/en.2011-1110 22045661 PMC3249677

[96] Clarke IJ Sari IP Qi Y Smith JT Parkington HC Ubuka T Potent action of RFamide-related peptide-3 on pituitary gonadotropes indicative of a hypophysiotropic role in the negative regulation of gonadotropin secretion. Endocrinology (2008) 149:5811–21. 10.1210/en.2008-0575 18617613

[97] Decourt C Anger K Robert V Lomet D Bartzen-Sprauer J Caraty A No evidence that RFamide-related peptide 3 directly modulates LH secretion in the ewe. Endocrinology (2016) 157:1566–75. 10.1210/en.2015-1854 26862995

[98] Simonneaux V Ancel C. RFRP neurons are critical gatekeepers for the photoperiodic control of reproduction. Front Endocrinol (2012) 3:168. 10.3389/fendo.2012.00168 23264769 PMC3524517

[99] Simonneaux V Ancel C Poirel VJ Gauer F. Kisspeptins and RFRP-3 act in concert to synchronize rodent reproduction with seasons. Front Neurosci (2013) 7:22. 10.3389/fnins.2013.00022 23550229 PMC3581800

[100] Seymour AJ Scott V Augustine RA Bouwer GT Campbell RE Brown CH. Development of an excitatory kisspeptin projection to the oxytocin system in late pregnancy. J Physiol (2017) 595:825–38. 10.1113/JP273051 27589336 PMC5285723

[101] Salehi MS Khazali H Mahmoudi F Janahmadi M. Oxytocin intranasal administration affects neural networks upstream of GNRH neurons. J Mol Neurosci (2017) 62:356–62. 10.1007/s12031-017-0943-8 28664358

[102] McGowan BM Stanley SA Donovan J Thompson EL Patterson M Semjonous NM Relaxin-3 stimulates the hypothalamic-pituitary-gonadal axis. Am J Physiol Endocrinol Metab (2008) 295:E278–86. 10.1152/ajpendo.00028.2008 18492777 PMC2519759

[103] Tanaka M Iijima N Miyamoto Y Fukusumi S Itoh Y Ozawa H Neurons expressing relaxin 3/INSL 7 in the nucleus incertus respond to stress. Eur J Neurosci (2005) 21:1659–70. 10.1111/j.1460-9568.2005.03980.x 15845093

[104] Vida B Deli L Hrabovszky E Kalamatianos T Caraty A Coen CW Evidence for suprachiasmatic vasopressin neurones innervating kisspeptin neurones in the rostral periventricular area of the mouse brain: regulation by oestrogen. J Neuroendocrinol (2010) 22:1032–9. 10.1111/j.1365-2826.2010.02045.x 20584108

[105] Piet R Fraissenon A Boehm U Herbison AE. Estrogen permits vasopressin signaling in preoptic kisspeptin neurons in the female mouse. J Neurosci (2015) 35:6881–92. 10.1523/JNEUROSCI.4587-14.2015 25926463 PMC6605180

[106] Karsch FJ Goodman RL Legan SJ Feedback basis of seasonal breeding: test of an hypothesis. J Reprod Fertil (1980) 58:521–35. 10.1530/jrf.0.0580521 7191896

[107] Barrell GK Moenter SM Caraty A Karsch FJ. Seasonal changes of gonadotropin-releasing hormone secretion in the ewe. Biol Reprod (1992) 46:1130–5. 10.1095/biolreprod46.6.1130 1391310

[108] Turek FW. The interaction of the photoperiod and testosterone in regulating serum gonadotropin levels in castrated male hamsters. Endocrinology (1977) 101:1210–5. 10.1210/endo-101-4-1210 908271

[109] Karsch FJ Bittman EL Foster DL Goodman RL Legan SJ Robinson JE Neuroendocrine basis of seasonal reproduction. Recent Prog Horm Res (1984) 40:185–232. 6385166 10.1016/b978-0-12-571140-1.50010-4

[110] Smith JT. Sex steroid control of hypothalamic Kiss1 expression in sheep and rodents: comparative aspects. Peptides (2009) 30:94–102. 10.1016/j.peptides.2008.08.013 18789989

[111] Revel FG Saboureau M Masson-Pevet M Pevet P Mikkelsen JD Simonneaux V. Kisspeptin mediates the photoperiodic control of reproduction in hamsters. Curr Biol (2006) 16:1730–5. 10.1016/j.cub.2006.07.025 16950111

[112] Singh SR Hileman SM Connors JM McManus CJ Coolen LM Lehman MN Estradiol negative feedback regulation by glutamatergic afferents to A15 dopaminergic neurons: variation with season. Endocrinology (2009) 150:4663–71. 10.1210/en.2009-0432 19589862 PMC2754677

[113] Goodman RL Maltby MJ Millar RP Hileman SM Nestor CC Whited B Evidence that dopamine acts via kisspeptin to hold GnRH pulse frequency in check in anestrous ewes. Endocrinology (2012) 153:5918–27. 10.1210/en.2012-1611 23038740 PMC3512065

[114] Mason AO Duffy S Zhao S Ubuka T Bentley GE Tsutsui K Photoperiod and reproductive condition are associated with changes in RFamide-related peptide (RFRP) expression in Syrian hamsters (*Mesocricetus auratus*). J Biol Rhythms (2010) 25:176–85. 10.1177/0748730410368821 20484689 PMC3266107

[115] Greives TJ Kriegsfeld LJ Demas GE Exogenous kisspeptin does not alter photoperiod-induced gonadal regression in Siberian hamsters (*Phodopus sungorus*). Gen Comp Endocrinol (2008) 156:552–8. 10.1016/j.ygcen.2008.02.017 18405899 PMC2430753

[116] Li XF Knox AM OByrne KT. Corticotrophin-releasing factor and stress-induced inhibition of the gonadotrophin-releasing hormone pulse generator in the female. Brain Res (2010) 1364:153–63. 10.1016/j.brainres.2010.08.036 20727865

[117] Rivier C Rivest S. Effect of stress on the activity of the hypothalamic-pituitary-gonadal axis: peripheral and central mechanisms. Biol Reprod (1991) 45:523–32. 10.1095/biolreprod45.4.523 1661182

[118] Tilbrook AJ Canny BJ Stewart BJ Serapiglia MD Clarke IJ Central administration of corticotrophin releasing hormone but not arginine vasopressin stimulates the secretion of luteinizing hormone in rams in the presence and absence of testosterone. J Endocrinol (1999) 162:301–11. 10.1677/joe.0.1620301 10425469

[119] Caraty A Miller DW Delaleu B Martin GB. Stimulation of LH secretion in sheep by central administration of corticotrophin-releasing hormone. J Reprod Fertil (1997) 111:249–57. 10.1530/jrf.0.1110249 9462293

[120] MacLusky NJ Naftolin F Leranth C. Immunocytochemical evidence for direct synaptic connections between corticotrophin-releasing factor (CRF) and gonadotrophin-releasing hormone (GnRH)-containing neurons in the preoptic area of the rat. Brain Res (1988) 439:391–5. 10.1016/0006-8993(88)91501-6 3282601

[121] Dudas B Merchenthaler I. Close juxtapositions between luteinizing hormone-releasing hormone-immunoreactive neurons and corticotropin-releasing factor-immunoreactive axons in the human diencephalon. J Clin Endocrinol Metab (2002) 87:5778–84. 10.1210/jc.2002-020996 12466386

[122] Hahn JD Kalamatianos T Coen CW. Studies on the neuroanatomical basis for stress-induced oestrogen-potentiated suppression of reproductive function: evidence against direct corticotropin-releasing hormone projections to the vicinity of luteinizing hormone-releasing hormone cell bodies in female rats. J Neuroendocrinol (2003) 15:732–42. 10.1046/j.1365-2826.2003.01056.x 12834433

[123] Li XF Mitchell JC Wood S Coen CW Lightman SL OByrne KT. The effect of oestradiol and progesterone on hypoglycaemic stress-induced suppression of pulsatile luteinizing hormone release and on corticotropin-releasing hormone mRNA expression in the rat. J Neuroendocrinol (2003) 15:468–76. 10.1046/j.1365-2826.2003.01014.x 12694372

[124] Rivest S Rivier C. Influence of the paraventricular nucleus of the hypothalamus in the alteration of neuroendocrine functions induced by intermittent footshock or interleukin. Endocrinology (1991) 129:2049–57. 10.1210/endo-129-4-2049 1655391

[125] Takumi K Iijima N Higo S Ozawa H. Immunohistochemical analysis of the colocalization of corticotropin-releasing hormone receptor and glucocorticoid receptor in kisspeptin neurons in the hypothalamus of female rats. Neurosci Lett (2012) 531:40–5. 10.1016/j.neulet.2012.10.010 23069671

[126] Tal R Taylor HS Burney RO Mooney SB Giudice LC Endocrinology of pregnancy. In: De Groot LJ Chrousos G Dungan K Feingold KR Grossman A Hershman JM, editors. Endotext. South Dartmouth, MA: MDText.com, Inc. (2000). Available from: https://www.ncbi.nlm.nih.gov/books/NBK278962/

[127] Reid RL Ling N Yen SS. Gonadotropin-releasing activity of alpha-melanocyte-stimulating hormone in normal subjects and in subjects with hypothalamic-pituitary dysfunction. J Clin Endocrinol Metab (1984) 58:773–7. 10.1210/jcem-58-5-773 6423658

[128] Roa J Herbison AE. Direct regulation of GnRH neuron excitability by arcuate nucleus POMC and NPY neuron neuropeptides in female mice. Endocrinology (2012) 153:5587–99. 10.1210/en.2012-1470 22948210

[129] Celis ME. Release of LH in response to alpha-MSH administration. Acta Physiol Pharmacol Latinoam (1985) 35:281–90. 2938412

[130] Scimonelli T Celis ME. A central action of alpha-melanocyte-stimulating hormone on serum levels of LH and prolactin in rats. J Endocrinol (1990) 124:127–32. 10.1677/joe.0.1240127 1967627

[131] Backholer K Smith JT Rao A Pereira A Iqbal J Ogawa S Kisspeptin cells in the ewe brain respond to leptin and communicate with neuropeptide Y and proopiomelanocortin cells. Endocrinology (2010) 151:2233–43. 10.1210/en.2009-1190 20207832

[132] Fu LY van den Pol AN. Kisspeptin directly excites anorexigenic proopiomelanocortin neurons but inhibits orexigenic neuropeptide Y cells by an indirect synaptic mechanism. J Neurosci (2010) 30:10205–19. 10.1523/JNEUROSCI.2098-10.2010 20668204 PMC2933146

[133] True C Verma S Grove KL Smith MS. Cocaine- and amphetamine-regulated transcript is a potent stimulator of GnRH and kisspeptin cells and may contribute to negative energy balance-induced reproductive inhibition in females. Endocrinology (2013) 154:2821–32. 10.1210/en.2013-1156 23736294 PMC3713223

[134] Manfredi-Lozano M Roa J Ruiz-Pino F Piet R Garcia-Galiano D Pineda R Defining a novel leptin-melanocortin-kisspeptin pathway involved in the metabolic control of puberty. Mol Metab (2016) 5:844–57. 10.1016/j.molmet.2016.08.003 27688998 PMC5034608

[135] Padilla SL Qiu J Nestor CC Zhang C Smith AW Whiddon BB AgRP to Kiss1 neuron signaling links nutritional state and fertility. Proc Natl Acad Sci U S A (2017) 114:2413–8. 10.1073/pnas.1621065114 28196880 PMC5338482

[136] Israel DD Sheffer-Babila S de Luca C Jo YH Liu SM Xia Q Effects of leptin and melanocortin signaling interactions on pubertal development and reproduction. Endocrinology (2012) 153:2408–19. 10.1210/en.2011-1822 22408174 PMC3381095

[137] Campbell RE ffrench-Mullen JM Cowley MA Smith MS Grove KL. Hypothalamic circuitry of neuropeptide Y regulation of neuroendocrine function and food intake via the Y5 receptor subtype. Neuroendocrinology (2001) 74:106–19. 10.1159/000054676 11474218

[138] Castellano JM Navarro VM Fernandez-Fernandez R Roa J Vigo E Pineda R Expression of hypothalamic KiSS-1 system and rescue of defective gonadotropic responses by kisspeptin in streptozotocin-induced diabetic male rats. Diabetes (2006) 55:2602–10. 10.2337/db05-1584 16936210

[139] Smith JT Acohido BV Clifton DK Steiner RA. KiSS-1 neurones are direct targets for leptin in the ob/ob mouse. J Neuroendocrinol (2006) 18:298–303. 10.1111/j.1365-2826.2006.01417.x 16503925

[140] Gonzalez LC Pinilla L Tena-Sempere M Aguilar E. Leptin(116-130) stimulates prolactin and luteinizing hormone secretion in fasted adult male rats. Neuroendocrinology (1999) 70:213–20. 10.1159/000054479 10516485

[141] Cravo RM Margatho LO Osborne-Lawrence S Donato J Jr Atkin S Bookout AL Characterization of Kiss1 neurons using transgenic mouse models. Neuroscience (2010) 173:37–56. 10.1016/j.neuroscience.2010.11.022 21093546 PMC3026459

[142] Cravo RM Frazao R Perello M Osborne-Lawrence S Williams KW Zigman JM Leptin signaling in Kiss1 neurons arises after pubertal development. PLoS One (2013) 8:e58698. 10.1371/journal.pone.0058698 23505551 PMC3591417

[143] Harrison JL Miller DW Findlay PA Adam CL. Photoperiod influences the central effects of ghrelin on food intake, GH and LH secretion in sheep. Neuroendocrinology (2008) 87:182–92. 10.1159/000112480 18073457

[144] Martini AC Fernandez-Fernandez R Tovar S Navarro VM Vigo E Vazquez MJ Comparative analysis of the effects of ghrelin and unacylated ghrelin on luteinizing hormone secretion in male rats. Endocrinology (2006) 147:2374–82. 10.1210/en.2005-1422 16455774

[145] Kluge M Schussler P Schmidt D Uhr M Steiger A. Ghrelin suppresses secretion of luteinizing hormone (LH) and follicle-stimulating hormone (FSH) in women. J Clin Endocrinol Metab (2012) 97:E448–51. 10.1210/jc.2011-2607 22259063

[146] Zigman JM Jones JE Lee CE Saper CB Elmquist JK. Expression of ghrelin receptor mRNA in the rat and the mouse brain. J Comp Neurol (2006) 494:528–48. 10.1002/cne.20823 16320257 PMC4524499

[147] Frazao R Dungan Lemko HM da Silva RP Ratra DV Lee CE Williams KW Estradiol modulates Kiss1 neuronal response to ghrelin. Am J Physiol Endocrinol Metab (2014) 306:E606–14. 10.1152/ajpendo.00211.2013 24473434 PMC3948981

[148] Clegg DJ Brown LM Zigman JM Kemp CJ Strader AD Benoit SC Estradiol-dependent decrease in the orexigenic potency of ghrelin in female rats. Diabetes (2007) 56:1051–8. 10.2337/db06-0015 17251274

[149] Chen R Wu X Jiang L Zhang Y. Single-cell RNA-Seq reveals hypothalamic cell diversity. Cell Rep (2017) 18:3227–41. 10.1016/j.celrep.2017.03.004 28355573 PMC5782816

[150] Custo Greig LF Woodworth MB Galazo MJ Padmanabhan H Macklis JD. Molecular logic of neocortical projection neuron specification, development and diversity. Nat Rev Neurosci (2013) 14:755–69. 10.1038/nrn3586 24105342 PMC3876965

[151] Toledo-Rodriguez M Blumenfeld B Wu C Luo J Attali B Goodman P Correlation maps allow neuronal electrical properties to be predicted from single-cell gene expression profiles in rat neocortex. Cereb Cortex (2004) 14:1310–27. 10.1093/cercor/bhh092 15192011

[152] Kokay IC Petersen SL Grattan DR. Identification of prolactin-sensitive GABA and kisspeptin neurons in regions of the rat hypothalamus involved in the control of fertility. Endocrinology (2011) 152:526–35. 10.1210/en.2010-0668 21177834

[153] Liu X Herbison A. Kisspeptin regulation of arcuate neuron excitability in kisspeptin receptor knockout mice. Endocrinology (2015) 156:1815–27. 10.1210/en.2014-1845 25756309

[154] Mayer C Boehm U. Female reproductive maturation in the absence of kisspeptin/GPR54 signaling. Nat Neurosci (2011) 14:704–10. 10.1038/nn.2818 21516099

[155] Weber T Bohm G Hermann E Schutz G Schonig K Bartsch D. Inducible gene manipulations in serotonergic neurons. Front Mol Neurosci (2009) 2:24. 10.3389/neuro.02.024.2009 19936315 PMC2779094

[156] Cong L Ran FA Cox D Lin S Barretto R Habib N Multiplex genome engineering using CRISPR/Cas systems. Science (2013) 339:819–23. 10.1126/science.1231143 23287718 PMC3795411

[157] Tervo DG Hwang BY Viswanathan S Gaj T Lavzin M Ritola KD A designer AAV variant permits efficient retrograde access to projection neurons. Neuron (2016) 92:372–82. 10.1016/j.neuron.2016.09.021 27720486 PMC5872824

[158] Swiech L Heidenreich M Banerjee A Habib N Li Y Trombetta J In vivo interrogation of gene function in the mammalian brain using CRISPR-Cas9. Nat Biotechnol (2015) 33:102–6. 10.1038/nbt.3055 25326897 PMC4492112

[159] Kobbert C Apps R Bechmann I Lanciego JL Mey J Thanos S. Current concepts in neuroanatomical tracing. Prog Neurobiol (2000) 62:327–51. 10.1016/S0301-0082(00)00019-8 10856608

[160] Swanson LW. Cerebral hemisphere regulation of motivated behavior. Brain Res (2000) 886:113–64. 10.1016/S0006-8993(00)02905-X 11119693

[161] Yeo SH Herbison AE. Projections of arcuate nucleus and rostral periventricular kisspeptin neurons in the adult female mouse brain. Endocrinology (2011) 152:2387–99. 10.1210/en.2011-0164 21486932

[162] Ugolini G. Advances in viral transneuronal tracing. J Neurosci Methods (2010) 194:2–20. 10.1016/j.jneumeth.2009.12.001 20004688

[163] Callaway EM Luo L Monosynaptic circuit tracing with glycoprotein-deleted rabies viruses. J Neurosci (2015) 35:8979–85. 10.1523/JNEUROSCI.0409-15.2015 26085623 PMC4469731

[164] Helmstaedter M. Cellular-resolution connectomics: challenges of dense neural circuit reconstruction. Nat Methods (2013) 10:501–7. 10.1038/nmeth.2476 23722209

[165] Wickersham IR Lyon DC Barnard RJ Mori T Finke S Conzelmann KK Monosynaptic restriction of transsynaptic tracing from single, genetically targeted neurons. Neuron (2007) 53:639–47. 10.1016/j.neuron.2007.01.033 17329205 PMC2629495

[166] Nectow AR Moya MV Ekstrand MI Mousa A McGuire KL Sferrazza CE Rapid molecular profiling of defined cell types using viral TRAP. Cell Rep (2017) 19:655–67. 10.1016/j.celrep.2017.03.048 28423326 PMC5476221

[167] Nectow AR Schneeberger M Zhang H Field BC Renier N Azevedo E Identification of a brainstem circuit controlling feeding. Cell (2017) 170:429–42.e11. 10.1016/j.cell.2017.06.045 28753423

[168] Aston-Jones G Deisseroth K Recent advances in optogenetics and pharmacogenetics. Brain Res (2013) 1511:1–5. 10.1016/j.brainres.2013.01.026 23422677 PMC3663045

[169] Arrigoni E Saper CB. What optogenetic stimulation is telling us (and failing to tell us) about fast neurotransmitters and neuromodulators in brain circuits for wake-sleep regulation. Curr Opin Neurobiol (2014) 29:165–71. 10.1016/j.conb.2014.07.016 25064179 PMC4268002

[170] Hill JW Elmquist JK Elias CF Hypothalamic pathways linking energy balance and reproduction. Am J Physiol Endocrinol Metab (2008) 294:E827–32. 10.1152/ajpendo.00670.2007 18285524 PMC5724360

[171] Foradori CD Coolen LM Fitzgerald ME Skinner DC Goodman RL Lehman MN. Colocalization of progesterone receptors in parvicellular dynorphin neurons of the ovine preoptic area and hypothalamus. Endocrinology (2002) 143:4366–74. 10.1210/en.2002-220586 12399433

[172] Burke MC Letts PA Krajewski SJ Rance NE. Coexpression of dynorphin and neurokinin B immunoreactivity in the rat hypothalamus: morphologic evidence of interrelated function within the arcuate nucleus. J Comp Neurol (2006) 498:712–26. 10.1002/cne.21086 16917850

